# Physiological Adaptation Strategies of the Interaction Defense Between Larvae of *Megastigmus sabinae* and the Host *Juniperus przewalskii*

**DOI:** 10.3390/insects17010124

**Published:** 2026-01-21

**Authors:** Huike Yao, Jianxin Zeng, Yahui Li, Dong Lv, Min Chen

**Affiliations:** 1 Beijing Key Laboratory for Forest Pest Control, Beijing Forestry University, Beijing 100083, China; huikyao0921@163.com (H.Y.);; 2 Gansu Province Academy of Qilian Water Resource Conservation Forests Research Institute, Zhangye 734000, China

**Keywords:** chemical defenses, defense strategies, *Juniperus przewalskii*, *Megastigmus sabinae*, metabolomics, synergistic development

## Abstract

*Juniperus przewalskii* Komarov, an endemic conifer in the high-altitude mountains of the Qinghai–Tibet Plateau, develops its cones in a synergistic manner with the oligophagous pest *Megastigmus sabinae* Xu et He (Hymenoptera: Torymidae), forming a highly specialized interaction system. However, the physiological adaptation mechanisms underlying this interaction remain unclear. Feeding by *M. sabinae* larvae significantly induced defense responses in *J. przewalskii* cones. During the early instars (2nd~3rd) of *M. sabinae* larvae, infested *J. przewalskii* endosperms upregulated protein content (48.91%; 3rd instar), significantly enhanced peroxidase (POD) activity (71.10%; 2nd instar), and specifically enriched coumarins and cinnamaldehyde derivatives (2nd instar) to increase *M. sabinae* larvae metabolic costs. In later instars (4th~5th) of *M. sabinae* larvae, the infested endosperms downregulated starch content (29.69%; 4th instar), increased phenylalanine ammonia-lyase (PAL) activity (57.34%; 4th instar), and accumulated steroid derivatives to suppress larvae development. Conversely, *M. sabinae* larvae demonstrated unique adaptive strategies: maintaining high levels of glutathione S-transferase (GST) as an antioxidant defense system during early instars and upregulating the level of digestive enzymes in later stages to overcome host multi-layered defenses. *Juniperus przewalskii* counters pest infestation through dynamic nutrient modulation, temporal activation of protective enzymes, and a multi-layered chemical defense network. The adaptation of *M. sabinae* larvae appears to involve the developmental regulation of detoxification and digestive enzyme levels. This study provides novel insights that enrich coevolution theory in alpine ecosystems.

## Simple Summary

The Tibetan Plateau’s unique conifer, *Juniperus przewalskii*, faces a major threat from a specialist insect, the parasitic wasp *Megastigmus sabinae*, whose larvae develop synergistically with the tree’s cones. Understanding how the tree defends itself and how the insect counteracts these defenses is crucial. Our study reveals that the tree deploys a dynamic, stage-specific defense strategy. When the larvae are young, the tree increases specific proteins and defensive compounds to raise the larvae’s metabolic costs. As the larvae grow, the tree instead reduces nutrient content and accumulates steroid-like substances to suppress their development. Remarkably, the insect larvae fight back effectively. They activate their detoxification system early on to neutralize plant toxins and later boost their digestive power to cope with the host’s nutrient limitations. This intricate back-and-forth illustrates a sophisticated arms race. Our findings provide new insights into the resilience of alpine ecosystems and can inform the development of sustainable forest management strategies that leverage these natural plant–insect interactions.

## 1. Introduction

Within forest ecosystems, the interaction between host plants and phytophagous insects remains a central theme in forest protection research. *Juniperus przewalskii* Komarov, an endemic alpine conifer species of the cypress family Cupressaceae (genus *Juniperus*) on the northeastern margin of the Qinghai–Tibet Plateau [1], exhibits a geographically restricted distribution confined to high-altitude mountains (2600~4300 m) ranging from the northeastern Qinghai–Tibet Plateau to the western edge of the Loess Plateau [2]. This species adapts to extreme drought and low-temperature environments through traits such as a deep root system, scaly leaf structure, and the accumulation of secondary metabolites (e.g., flavonoids) [3,4]. It serves as a dominant constructive species within the montane forest-steppe ecotone [5,6]. Its cones provide the exclusive reproductive substrate for the juniper seed pest, *Megastigmus sabinae* Xu et He (Hymenoptera: Torymidae), establishing a highly specialized parasitic relationship. This interaction system offers an ideal model for investigating plant–insect interaction and deciphering the spatiotemporal dynamics of host plant defenses versus insect counter-adaptations [7]. Recent studies demonstrate that plants employ secondary metabolites as a key strategy in constructing chemical defense systems against seed and cone pests [8,9], while insects develop detoxification mechanisms, often involving metabolic plasticity, to overcome these plant defenses [10]. However, our understanding of high-altitude juniper-pest systems remains limited. Specifically, the spatiotemporal coupling between dynamic defense strategies during cone development and insect counter-defense mechanisms remains unclear, as does the interaction between the dynamics of secondary metabolites in high-altitude Cupressaceae plants and the diverse enzyme activities of their insect pests.

*Megastigmus sabinae* and *Juniperus przewalskii* exhibit a highly specific synergistic developmental relationship. Adults oviposit into healthy cones, and the larval stage, comprising five instars, extends from late August of the current year to late June of the following year. The majority of larvae overwinter predominantly as 2nd instar (with a minority overwintering as 1st or 3rd instars) [11]. However, the development of insects is often influenced by the prevailing and local climatic conditions. Larvae from the 1st to 4th instars bore into the endosperm, their development synchronizes with that of the host cones, culminating in the pupal stage, coinciding with the complete depletion of endosperm nutrients, resulting in hollow and abortion of cones [12]. This protracted developmental period and synchrony render the larvae entirely dependent on cone resources throughout their development. However, it also exacerbates seed abortion in *J. przewalskii* due to the barrier to natural regeneration. Current research primarily focuses on the biological characteristics and control techniques of *M. sabinae* [13]. Nevertheless, the physiological adaptation mechanisms underlying its interaction with the host plant remain unclear.

Plant secondary metabolites, such as flavonoids, coumarins, and steroidal compounds, play crucial roles in defending against feeding by herbivorous insects. Flavonoids exert insecticidal effects by inducing cytotoxicity or forming complexes with various enzymes, thereby impairing insect behavior and growth [14,15]. *Picea* spp. defend against *Pissodes strobi* larvae by activating terpene-rich oleoresin chemical defenses and increasing the abundance of stone cells [16]. This synergistic physical and chemical defense mechanism is prevalent in conifer cone defense mechanisms. However, systematic investigation into the temporal dynamics of secondary metabolites in Cupressaceae plants, particularly their correspondence with insect stages, remains lacking.

Research on insect adaptation strategies to plant defenses shows a multidimensional trend. Biochemically, insects rely on detoxifying enzyme systems, such as cytochrome P450 monooxygenases (CYP450), glutathione S-transferases (GST), and carboxylesterases (CarE), to degrade plant antiherbivore chemical defenses [17,18]. Physiologically, they dynamically regulate digestive enzymes to cope with host nutrient limitations, enhancing physiological plasticity [19]. From a genetic evolutionary perspective, certain oligophagous groups develop specific adaptation mechanisms through key gene mutations—for instance, oligophagous species within the noctuid genus *Spodoptera* cannot feed on Poaceae plants containing the toxin DIMBOA (2,4-dihydroxy-7-methoxy-2H-1,4-benzoxazin-3(4H)-one) due to mutations of UGT genes (UDP-glycosyltransferases) [20]. However, current research predominantly focuses on polyphagous Lepidoptera species [20,21,22], with a paucity of systematic analysis on defense mechanisms in oligophagous Hymenoptera species. For example, *M. sabinae* larvae complete their entire larval stage within the cones of *J. przewalskii*, likely evolving unique physiological adaptation mechanisms and detoxification metabolic pathways. The molecular basis and ecological significance of these adaptations warrant further exploration.

In summary, while research on the physiological defense mechanisms of conifer seed pests and the corresponding counter-defense adaptation strategies of larvae has made progress, most studies have focused on interaction systems composed of Pinaceae *Picea* spp. plants and Coleoptera Curculionidae insects [16,23,24]. In contrast, there has been a lack of attention to the interaction systems formed by Cupressaceae plants and their specialized parasitic insects. Furthermore, existing research has predominantly analyzed either plant chemical defenses (metabolomics) or insect detoxification mechanisms (enzyme activities and transcriptomics) in isolation, lacking an integrated analysis of both within the same developmental cycle. There is a particular scarcity of studies that simultaneously analyze the multi-layered defensive responses (nutrition, protective enzymes and metabolomics) during host cone development and the linked changes in the multi-enzyme systems of corresponding larval instars of the pest. Therefore, illustrating the bidirectional and dynamic physiological adaptation strategies within such an interaction system is crucial.

Based on this background, our study for the first time establishes a multi-level interaction system between a typical high-altitude tree species (*J. przewalskii*) and its oligophagous pest (*M. sabinae*), integrating nutrient content analysis, metabolomic analysis, and multi-category enzyme level profiling. Primarily, we analyzed dynamic nutrient content and protective enzyme activities in cones following parasitism by *M. sabinae* larvae. Subsequently, based on liquid chromatography-mass spectrometry (LC-MS) technology, we analyzed differential secondary metabolites in infested cones in response to larvae feeding. Finally, we determined the digestive and detoxifying enzyme level of larvae during their synergistic development within the cones. This study aims to address the following scientific questions: (1) How do infested cones establish multi-layered defense through nutrient content remodeling, responsive regulation of protective enzyme activities, and temporal accumulation of secondary metabolites? (2) How do larvae achieve metabolic adaptation through dynamic modulation of digestive and detoxifying enzymes? (3) What are the quantitative coupling relationships between cone defense strategies and larval adaptation mechanisms? This work lays the foundation for elucidating the physiological adaptation strategies underpinning nutritional and chemical defense interactions formed during the long-term coevolutionary process between *J. przewalskii* and *M. sabinae*.

## 2. Materials and Methods

### 2.1. Insect Material and Host Plant Cones

Mature cones of *Juniperus przewalskii* were collected from the forest stand in Toutan, Sunan Yugur Autonomous County, Zhangye City, Gansu Province (altitude: 2920 m; 38°32′ N, 100°14′ E). *Megastigmus sabinae* insects were collected on five dates. The specific collection dates and their corresponding developmental stages of *M. sabinae* are shown in Table 1. For each collection, cones were collected from 50 individual trees, with 1000 cones sampled for each larval instar. The collected cones were immediately transported to the laboratory, longitudinally bisected, and examined to distinguish healthy cones from infested ones. Both cone material and larvae from each stage were immediately frozen and stored at −80 °C for subsequent analysis. For subsequent biochemical and metabolomic analysis, cones collected from all 50 trees for a given date and infestation status were pooled. Biological replicates were then created by randomly drawing sub-samples from this pooled population. This approach aims to measure the average response of the tree population while acknowledging that it integrates variation across individual trees.

In the laboratory, cones stored at −80 °C were retrieved, longitudinally sectioned under a microscope. After distinguishing healthy and damaged cones, the cones were dissected, and the development status of the cones and *M. sabinae* was observed using a biological microscope (Leica M205 FA, Wetzlar, Germany), and then photographed and measured using ZEN (v3.10, Carl Zeiss (Shanghai) Management Co., Ltd., Shanghai, China). The criteria for determining the larval instar of *M. sabinae* refer to Lv et al. [11]. Specifically, for each dissected larvae, four morphological variables were measured: maxillary joint width, head width, body width, and body length. The mean value, standard error, coefficient of variation, Brooks index, and Crosby index of these four variables were calculated, and the larvae were divided into five instars. Furthermore, given that *M. sabinae* larvae primarily feed on the endosperm of the cones, the endosperm tissue isolated from the cones served as the uniform experimental material for all correlation analysis in this study. Specifically, in the laboratory, after the cones were longitudinally sectioned, they were further dissected to isolate and collect the endosperm tissue for subsequent experiments.

### 2.2. Nutrient Contents Analysis in Juniperus przewalskii Endosperms

#### 2.2.1. Protein Content

Protein content in healthy and infested *J. przewalskii* endosperms across developmental stages was quantified using the BCA Protein Assay Kit (Beijing Bairuiji Biotechnology Co., Ltd., Beijing, China), according to the manufacturer’s instructions. Each experimental group included three biological replicates. Finally, using the function formula of the standard curve to calculate the protein concentration of each sample.

#### 2.2.2. Starch Content

Starch content in healthy and infested *J. przewalskii* endosperms across developmental stages was quantified using the Plant Starch Content Kit (Nanjing Jiancheng Bioengineering Institute, Nanjing, China), according to the manufacturer’s instructions. Each experimental group included three biological replicates. Starch content was calculated using the following formula:

(1) $$Starch\;content=\frac{A_{determination}-A_{blank}}{A_{standard}-A_{blank}}\times C_{standard}\times V_{sample}\times \frac{N}{W}$$

where *C_standard_* refers to the concentration of the Standard Solution, *V_sample_* is the total volume of the sample, *N* is the dilution factor of the supernatant, and *W* is the fresh weight of the sample.

#### 2.2.3. Crude Fat Content

Crude fat content of *J. przewalskii* endosperms was determined by Soxhlet extractor method, which was invented by German scientist Franz Ritter von Soxhlet in 1879. First, about 1 g of healthy or infested endosperms was weighed and placed on the filter paper, wrapped, and fixed with cotton thread before marking. Then, the filter paper was put into the extraction bag, and the various parts of the instrument were assembled. Two thirds of petroleum (mL) ether was added to the receiver, and heated under the condition of 60~75 °C water bath, so that the petroleum ether was continuously refluxed and extracted eight times h^−1^ for 6~8 h. Next, the receiving bottle was taken off, and the petroleum ether was recovered. When the residual petroleum ether in the receiving bottle was only 1~2 mL, it was evaporated in a water bath, and then dried in an oven at 105 ± 5 °C for 2 h, until the drying device was cooled and weighed. Each experimental group included three biological replicates. Finally, crude fat contents of each sample were calculated by the formula following:

(2) $$Crude\;fat\;content=\frac{{m_{1}-m}_{0}}{m_{2}}\;\times 100$$

where *m*_1_ refers to the quality of the receiving bottle and crude fat, *m*_0_ is the quality of the receiving bottle, and *m*_2_ is the quality of each sample.

### 2.3. Protective Enzyme Activity Analysis in Juniperus przewalskii Endosperms

#### 2.3.1. Peroxidase Activity

POD activity in healthy and infested *J. przewalskii* endosperms across stages was quantified using the Peroxidase Assay Kit (Nanjing Jiancheng Bioengineering Institute, Nanjing, China), according to the manufacturer’s instructions. Each experimental group included three biological replicates. Peroxidase activity was calculated using the following formula, the activity of an enzyme was shown as U per g of fresh weight:

(3) $$POD\;Activity=\frac{A_{determination}+A_{blank}}{12\times d}\times \frac{V_{RS}}{V_{sample\;quality}}÷T÷\frac{W}{V_{S}}\times 1000$$

where *V_RS_* refers to the total volume of the reaction system, *V_Sample quality_* is the quality of the sample; *T* is the response time (i.e., 20 min), *W* is the fresh weight of the tissue, *V_S_* is the total volume of the sample.

#### 2.3.2. Phenylalanine Ammonia Lyase Activity

PAL activity in healthy and infested *J. przewalskii* endosperms across stages was quantified using the Phenylalanine ammonia lyase Kit (Nanjing Jiancheng Bioengineering Institute), according to the manufacturer’s instructions. Each experimental group included three biological replicates. Phenylalanine ammonia lyase activity was calculated using the following formula, the activity of an enzyme is shown as U per g of fresh weight:

(4) $$PAL\;Activity=\frac{\Delta A}{0.1}\;÷(\frac{W}{V_{CE}}\times VS)\times \frac{V_{RS}}{1}÷T$$

where *W* refers to the weight of the sample, *V_CE_* is the total volume of the crude extract, *V_S_* is the loading amount of the crude extract during determination, *V_RS_* is the total volume of the reaction system, *T* is the response time, Δ*A = A _determination_ − A _blank_*.

### 2.4. LC-MS-Based Untargeted Metabolomics and Differential Metabolite Screening in Juniperus przewalskii Endosperms

Metabolites in healthy and infested *J. przewalskii* endosperms were analyzed using liquid chromatography-mass spectrometry (LC-MS; Thermo Scientific Q Exactive HF-X, Waltham, MA, USA) in two batches due to prolonged intervals between larval stages. Cones collected on 18 December 2021 (i.e., 2nd instar), 5 April 2022 (i.e., 3rd instar), and 10 May 2022 (i.e., 4th instar) were longitudinally dissected to separate healthy and infested cones. This resulted in *n* = 5 biological replicates for the healthy group and *n* = 5 biological replicates for the infested group at each time point. Each biological replicate was independently processed through extraction. The LC-MS analysis for each extract was performed with three technical replicates to ensure measurement precision. Samples were flash-frozen in liquid nitrogen and homogenized, with specific procedures as follows. Briefly, 0.1 g of the *J. przewalskii* endosperm tissue was weighed and transferred into a 2 mL Eppendorf tube. Methanol containing *L-2-*chlorophenylalanine (4 mg kg^−1^; pre-cooled to −20 °C) (Merck KGaA, Darmstadt, Germany) was added for 30 s vortex oscillation. After fully mixing, 100 mg of glass beads were added to each centrifuge tube, then they were all placed in the tissue grinder (Thermo Fisher Scientific Inc., Waltham, MA, USA) and ground for 90 s at 60 Hz. Subsequently, ultrasonic treatment was performed at room temperature for 15 min, and centrifuged at 4 °C and 12,000 rpm for 10 min, the supernatant was filtered with a 0.22 μm membrane and detected by LC-MS. In addition, some of the extracted samples to be tested were mixed into quality control (QC) samples to evaluate the stability and repeatability of the experimental method, and the QC inspection was performed on more than 10 experimental samples. The samples were eluted and determined by reference to the method of Want et al. [25].

Raw LC-MS data were converted to mzXML format using ProteoWizard version 3.0.8789 and processed via XCMS for peak alignment and normalization. The data were then normalized using the LOESS (Locally Estimated Scatterplot Smoothing) signal correction method based on QC samples to eliminate systematic errors. Prior to multivariate statistical analysis, the normalized data were scaled using the Unit Variance scaling (UV scaling) method. Metabolites were annotated using the Human Metabolome Database (HMDB, v5.0, https://hmdb.ca, accessed on 5 March 2024), MassBank (v2023.12, https://massbank.eu, accessed on 12 January 2024), Kyoto Encyclopedia of Genes and Genomes (KEGG, Release 107.0, https://www.kegg.jp, accessed on 31 January 2024), and LipidMaps (2024Q1 update, https://www.lipidmaps.org, accessed on 20 March 2024). Only metabolites with QC showing relative standard deviation (RSD) < 30% across technical replicates and detection rates > 80% in biological samples were retained. Subsequently, differential metabolites between healthy and infested endosperms were identified based on statistical significance (*p*-value), fold change, and the Variable Importance in Projection (VIP) score (VIP > 1 indicates that the variable’s contribution to class discrimination is above average), with multiple testing correction performed using the False Discovery Rate (FDR) method, specifically the Benjamini–Hochberg (BH) procedure. Following FDR correction, metabolites with an adjusted *p*-value < 0.05 and a VIP score > 1 were considered statistically significant differential metabolites.

### 2.5. Concentrations of Detoxification Enzymes in Megastigmus sabinae Larvae

First, to prepare the sample, larvae were collected from infested *J. przewalskii* cones at each developmental instar (i.e., 2nd, 3rd, 4th, and 5th). For each instar, a total of 50 larvae were pooled and randomly divided into five independent biological replicates, with each replicate consisting of 10 larvae. This resulted in a sample size of n = 5 biological replicates per group per instar. Each biological replicate was independently homogenized to obtain one tissue homogenate for subsequent enzyme-level measurement. Then, the pre-cooled sample was homogenized in a homogenizer, and the supernatant was taken for subsequent determination. In this study, the concentrations of CYP450, GST and CarE were determined to measure the detoxification enzyme level in the larvae of *M. Sabinae*. Enzyme-linked immunosorbent assay (ELISA) was used in the determination, and the specific steps were taken as the example of the determination of GST level.

The Insect Glutathione S-transferase ELISA Kit (Jingmei Biotechnology Co., Ltd., Nanjing, China) was used to determine the concentration of GST in the larvae of *M. Sabinae*. According to the manufacturer’s instructions, samples, standard and Horseradish Peroxidase (HRP) detection antibody were added to the micropores pre-covered with GST antibody, incubated at 37 °C for 1 h, and fully cleaned. Subsequently, under the action of TMB, the samples were first converted to blue with the catalyst of peroxidase, and last turned yellow; the color intensity was positively correlated with the concentration of GST in the samples. Finally, the stopping solution was added, and the absorbance (OD value) was determined by a microplate reader at 450 nm. The linear regression curve was drawn with the standard concentration as the abscissa and the OD value as the ordinate, and the concentration of each sample was quantified according to the curvilinear equation.

It should be noted that this ELISA quantifies the protein concentration of the target enzyme. In current research in physiology and biochemistry, measuring the concentration of enzyme is a widely accepted approach that shows a high correlation with enzyme activity assays, although it does not directly measure catalytic activity [19,26,27].

### 2.6. Concentrations of Digestive Enzymes in Megastigmus sabinae Larvae

The protease level, amylase level and lipase level of the larvae were determined to measure the digestive enzyme concentration in this study, also based on the ELISA. The specific procedures were the same as the detoxification-enzyme-level determination method above. The results are expressed in units per liter (U/L), which are derived from a standard curve constructed using serial dilutions of purified enzymes with pre-assayed catalytic activity.

### 2.7. Statistical Analysis

All statistical analyses were performed using IBM SPSS Statistics version 23.0 (IBM Corp., Armonk, NY, USA). Two-way analysis of variance (Two-way ANOVA) was employed to analyze the differences in nutrient contents of *J. przewalskii* endosperms and the activities of protective enzymes. For the detoxification and digestive enzyme levels of *M. sabinae* larvae, a one-way analysis of variance (ANOVA) was applied. Tukey’s honestly significant difference (HSD) post hoc test was used for multiple comparisons among different treatments to determine significant differences at the *p* < 0.05 level. Figures were generated using GraphPad Prism version 10.1.2 (GraphPad Software, San Diego, CA, USA).

## 3. Results

### 3.1. Nutrient Contents of Juniperus przewalskii Endosperms

**Figure 1:**
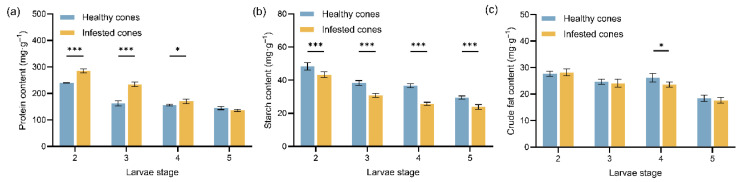
Mean values (±standard error) of the three major nutrient contents in healthy and infested endosperms of *Juniperus przewalskii* at different larval instars: (**a**) Protein content; (**b**) Starch content; (**c**) Crude fat content. Asterisks specify that the values were significantly different: *** *p* < 0.001, ** *p* < 0.01, * *p* < 0.05, Tukey’s multiple comparisons test.

The principal nutrient contents (protein, starch, and crude fat content) of healthy and infested endosperms of *J. przewalskii* across larval stages was determined (Figure 1). Overall, protein content was significantly higher than that of starch and crude fat, and all three nutrient contents exhibited a progressive decline in both healthy and infested endosperms over the developmental period. Infested endosperms consistently displayed higher protein content but lower starch content compared to healthy endosperms at all sampled stages, indicating larval feeding-induced alterations in internal nutrient composition. Specifically, infested endosperms exhibited significantly higher protein content than healthy endosperms during the earlier larvae instars (2nd~3rd instar; *p* < 0.001, F(3,16) = 358.40, η^2^ = 0.81; Figure 1a), while this difference diminished during later instars (4th~5th instar), showing only marginal (*p* < 0.05) or no significant differences (*p* > 0.05). Conversely, infested endosperms demonstrated significantly reduced starch content compared to healthy endosperms at every larval stage (*p* < 0.001, F(3,16) = 193.20, η^2^ = 0.76; Figure 1b). For crude fat content, no significant differences (*p* > 0.05, F(3,16) = 70.30, η^2^ = 0.90) were generally observed between infested and healthy endosperms, except for a slight decrease in infested endosperms during the 4th instar (Figure 1c).

### 3.2. Protective Enzyme Activity of Juniperus przewalskii Endosperms

**Figure 2:**
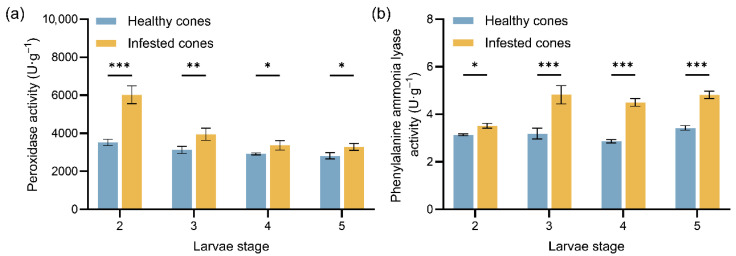
Mean values (±standard error) of protective enzyme activities in healthy and infested endosperms of *J. przewalskii* at different larval instars: (**a**) Peroxidase (POD) enzyme activity; (**b**) Phenylalanine ammonia lyase (PAL) enzyme activity. Asterisks specify that the values were significantly different: *** *p* < 0.001, ** *p* < 0.01, * *p* < 0.05, Tukey’s multiple comparisons test.

Larvae feeding by *M. sabinae* significantly altered the protective enzyme activities in *J. przewalskii* endosperms compared to healthy endosperms, with both POD and PAL activities exhibiting varying degrees of increase in infested endosperms (Figure 2). POD activity in healthy endosperms remained relatively stable throughout the developmental stages, ranging from 2811.12 to 3520.67 U/g. In contrast, POD activity in infested endosperms was significantly higher than in healthy endosperms during the earlier instars (2nd~3rd instar; F(3,16) = 106.50, η^2^ = 0.48). The most pronounced increase occurred during the 2nd instar, reaching 6280.39 U/g in infested endosperms, a 71.10% increase compared to healthy endosperms at the same stage (*p* < 0.001). Similarly, POD activity during the 3rd instar was 26.23% higher in infested endosperms (*p* < 0.01). PAL activity in healthy endosperms also remained stable across developmental stages, ranging from 2.86 to 3.43 U/g. Unlike the POD activity pattern where larval instar was the dominant factor, the health or infestation status had a highly significant main effect on endosperms PAL activity (*p* < 0.001, F(1,16) = 277.80, η^2^ = 0.68). Infested endosperms showed marginally higher PAL activity than healthy endosperms during the 2nd instar (*p* < 0.05). Subsequently, PAL activity in infested endosperms became highly significantly elevated during the 3rd to 5th instar larvae feeding periods, showing increases of 51.57%, 57.34%, and 42.37% compared to healthy endosperms, respectively.

### 3.3. Metabolomic Analysis Reveals Differential Metabolites in Juniperus przewalskii Endosperms

**Figure 3:**
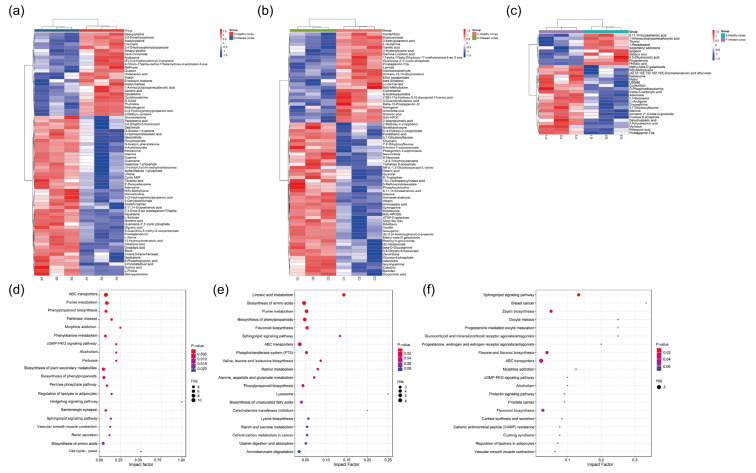
Differential metabolites (DMs) in *Juniperus przewalskii* endosperms between healthy cones and those infested by *Megastigmus sabinae* larvae at different larval instars. Hierarchical cluster analysis (HCA) between healthy and infested endosperms: (**a**) 2nd instar; (**b**) 3rd instar; (**c**) 4th instar. The color represents the level of metabolite content; the redder the color, the higher the content, and the bluer the color, the lower the content. KEGG pathway analysis of DMs between healthy and infested endosperms: (**d**) 2nd instar; (**e**) 3rd instar; (**f**) 4th instar. The color of the dots represents the level of enrichment, with redder indicating a more significant enrichment. The size of the dots represents the number of DMs annotated to the pathway.

Untargeted LC-MS-based metabolomic analysis was employed to profile secondary metabolites in healthy and infested *J. przewalskii* endosperms during the 2nd to 4th instar. A total of 437, 513, and 513 secondary metabolites were identified in healthy and infested endosperms during the 2nd, 3rd, and 4th instar, respectively (Figure 3).

Differential metabolite analysis between infested and healthy endosperms within each larval stage was performed. As shown in Figure 3, 73 differential metabolites (DMs) were identified during the 2nd instar (48 upregulated, 25 downregulated). Similarly, 73 DMs were identified during the 3rd instar (46 upregulated, 27 downregulated). In contrast, the number of DMs decreased to 36 during the 4th instar (25 upregulated, 11 downregulated). Comparative analysis of DMs across larval stages identified three key classes of defense-related secondary metabolites consistently enriched (high cumulative VIP scores) in multiple instars: carboxylic acids and derivatives, benzene and substituted derivatives, and flavonoids (Table 2). Notably, several identified DMs are known insect-resistance compounds. This finding indicates that feeding by *M. sabinae* significantly activates chemical defense responses in *J. przewalskii*, which persist across larval developmental stages. For instance, the enrichment of flavonoids suggests potential impairment of larval nutrient absorption efficiency via the flavonoid biosynthesis pathway. Furthermore, stage-specific DM enrichment was observed: coumarin (VIP = 6.2) and cinnamaldehyde derivatives (VIP = 5.8) were specifically enriched during the 2nd instar. Novel enrichment of steroid derivatives occurred during the 3rd and 4th instar larvae feeding periods; these compounds may enhance cone defense by disrupting insect hormonal balances.

KEGG enrichment analysis (Figure 3d–f) revealed that DAMs in infested endosperms were significantly enriched in pathways including Phenylpropanoid biosynthesis, ABC transporters, as well as pathways related to hormone signaling (e.g., Sphingolipid signaling pathway; Retinol metabolism) compared to healthy endosperms. This metabolic reprogramming suggests a potential multi-faceted response of *J. przewalskii* to infestation, involving secondary metabolism, transport processes, and modulation of hormone-related pathways.

### 3.4. Concentrations of Digestive Enzymes in Megastigmus sabinae Larvae

**Figure 4:**
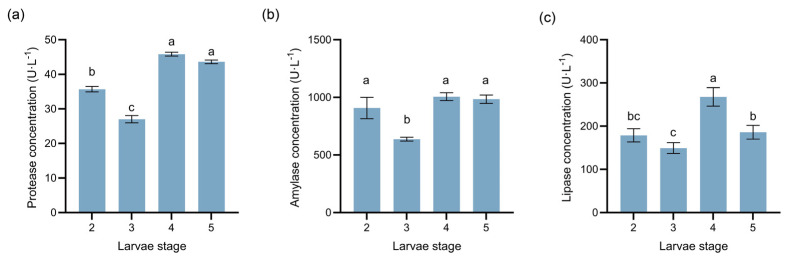
Dynamic changes in the concentrations of the three digestive enzymes at different instars of *Megastigmus sabinae* larvae: (**a**) Protease level, (**b**) Amylase level, (**c**) Lipase level. Different letters above the bars indicate significant differences: *p* < 0.05, as determined by one-way ANOVA and Tukey’s multiple comparisons test.

To assess larvae digestive adaptation to host plant nutrients, we measured the concentrations of protease, amylase, and lipase in *M. sabinae* larvae across the 2nd to 5th instars (Figure 4). Overall, larvae in later instars (4th~5th) exhibited significantly higher levels for all three digestive enzymes compared to those in earlier instars (2nd~3rd; Figure 4; *p* < 0.05). Protease level showed the most pronounced increase (62.96%; F(3,8) = 380.70, R^2^ = 0.99), with enzyme level generally increasing with larval stage. Lipase level demonstrated only a slight increase during the 5th instar (F(3,16) = 47.31, R^2^ = 0.90).

### 3.5. Concentrations of Detoxification Enzymes in Megastigmus sabinae Larvae

**Figure 5:**
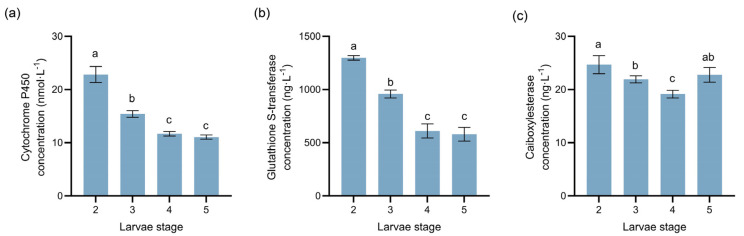
Dynamic changes in the concentrations of three detoxification enzyme at different instars of *M. sabinae* larvae: (**a**) Cytochrome P450 (CYP450) level, (**b**) Glutathione S-transferase (GST) level, (**c**) Carboxylesterase (CarE) level. Different letters above the bars indicate significant differences: *p* < 0.05, as determined by one-way ANOVA and Tukey’s multiple comparisons test.

To investigate larvae detoxification mechanisms in response to plant secondary metabolites in *J. przewalskii* cones, we measured the protein concentration of CYP450, GST, and CarE in *M. sabinae* larvae across the 2nd to 5th instars (Figure 5). The results revealed a consistent overall trend in the level of all three enzymes during larvae development. Significantly higher levels were observed in larvae of earlier instars (2nd~3rd) compared to those in later instars (4th~5th; *p* < 0.05). Generally, detoxification enzyme level decreased with the increase in larval stage (CYP450: (F(3,16) = 198.60, R^2^ = 0.97), GST: (F(3,16) = 215.40, R^2^ = 0.98)), except for CarE, which showed a slight increase during the 5th instar (F(3,16) = 18.36, R^2^ = 0.78).

## 4. Discussion

The interaction between phytophagous insects and their host plants represents a central theme in ecological interaction research [9]. Herbivory activates complex defense response networks in host plants [9], while insects counter these defenses through adaptive adjustments in developmental strategies and detoxification metabolic systems [28]. This study focuses on the unique interaction system between *Megastigmus sabinae* larvae and *Juniperus przewalskii* cones. Elucidating their multi-layered interaction mechanisms and physiological adaptation strategies provides novel insights into plant–insect co-defensive and adaptive evolutionary processes within high-altitude ecosystems.

This study reveals, for the first time, that *J. przewalskii* cones resist *M. sabinae* larvae feeding through the synergistic action of dynamically regulated nutrient composition and a multi-layered chemical defense network. Concurrently, shifts in multiple enzyme levels in *M. sabinae* larvae reflect their adaptive evolutionary responses to host plant defenses. Firstly, the protein content in infested endosperms was significantly higher than that in healthy endosperms, particularly during the early larvae instars (2nd~3rd). This likely results from *J. przewalskii* inducing the synthesis and accumulation of specific proteins to interfere with larvae’s overwintering survival and post-diapause digestion. For instance, infested cones exhibited significantly higher levels of defensive protective enzymes (e.g., peroxidases, polyphenol oxidases). These enzymes may interfere with the insect’s digestive process, reducing its digestive efficiency and increasing the larval metabolic cost [29]. This aligns with our observation that the levels of digestive enzymes (e.g., protease) are relatively low in early instars. Additionally, based on the significantly lower protease levels in early instars and insights from studies in other plant systems, we hypothesize that the defense response of *J. przewalskii* may involve the induction of protease inhibitors (PIs) and other defense-related proteins (e.g., pathogenesis-related proteins (PRs), chitinases) [30,31], which could restrict the potential activity of larval proteases. This hypothesis requires direct validation in future research through methods such as proteomics. In response, the maintenance of low protease levels by the larvae may represent an adaptive strategy to minimize these associated metabolic costs [32]. Secondly, starch content decreased significantly in infested endosperms. This decline may reflect its conversion to soluble sugars to compensate for carbohydrate depletion following larval feeding, aligning with a “nutrient limitation” strategy [33]. Compared to healthy endosperms, the reduced starch content in infested endosperms coincided with larvae amylase level being significantly higher than protease and lipase level. This suggests that the larvae may maintain higher levels of amylase to potentially enhance the efficiency of starch utilization, while keeping lower levels of lipase, which may help preserve lipid energy reserves during overwintering. This strategy is highly consistent with the ‘carbon-prioritizing and lipid-conserving strategy’ observed in gall-inducing insects during overwintering [34]. In high-altitude environments, carbon resources are particularly limited due to the short growing season and frequent drought stress, while the prolonged overwintering period imposes a stronger demand for the conservation of energy reserves (e.g., lipids). Analogous adaptations are seen in oligophagous seed pests like *Callosobruchus maculatus* and *Plodia interpunctella* [35,36], indicating oligophagous insects may overcome host nutrient limitations through metabolic plasticity. For example, *Eriogyna pyretorum* enhances trypsin activity when feeding on hosts low in soluble protein content to acquire more protein [19,37]. Thirdly, the decrease in crude fat content in infested cones, coupled with significantly elevated larval lipase level during the 4th instar and the enrichment of novel steroid derivatives during the 3rd~4th instar feeding period. This co-occurrence leads us to hypothesize a link where these steroid derivatives may interfere with insect lipid metabolism, for instance by inhibiting juvenile hormone synthesis—a testable hypothesis for future biochemical studies. Such interference could reduce available lipid nutrients for larvae, thereby delaying larval development [38]. This phenomenon also highlights the larvae’s adaptive feedback to such liposoluble toxins.

Furthermore, the temporal pattern of protective enzyme activity in cones indicates a ‘developmental stage-specific division of labor’ strategy. During the overwintering period (2nd instar), peroxidase (POD) activity was significantly elevated in infested endosperms. This likely serves to scavenge reactive oxygen species (ROS) accumulated prior to overwintering, induced by mechanical damage and insect saliva, thereby maintaining cellular homeostasis [39,40]. Analogously, *Bemisia tabaci* (Gennadius) feeding significantly induces enhanced POD activity in *Vigna mungo* (L.) Hepper. As larval development progressed (3rd~5th instars), phenylalanine ammonia-lyase (PAL) activity significantly increased. This observation is consistent with our LC-MS analysis: during this period, the phenylpropanoid pathway was significantly enriched in infested endosperms (Figure 3e), and multiple flavonoid and coumarin differential metabolites were identified (Table 1). These findings indicate that the rise in PAL activity likely drives the sustained activation of the phenylpropanoid pathway, thereby promoting the accumulation of such defense compounds.

The protein levels of key detoxification enzymes (CYP450, GST, and CarE) in the larvae revealed distinct developmental-stage-specific patterns: the levels of all three enzymes peaked during the early instar (2nd instar), followed by a significant decline as development advanced to later instars (4th~5th). This closely correlates with changes in secondary metabolites within infested endosperms; our data show (Figure 3a) that during the early larval stage (2nd instar), various secondary metabolites (e.g., coumarins and cinnamaldehyde derivatives) were markedly enriched, which coincided with the high levels of detoxification enzyme in the larvae at this period (Figure 5). This supports a reasonable hypothesis that the larvae may upregulate the biosynthesis of these detoxification enzymes to prepare for confronting the diverse secondary metabolites enriched in this stage. By the later instar (4th), larvae have largely adapted to the host cone environment, shifting focus towards accelerated growth and development. Concurrently, the variety of secondary metabolites upregulated by the host cones during the later larval stage was markedly reduced (25 types). Consistent with this adaptive shift, the protein levels of digestive enzymes in later instar larvae (4th~5th) were significantly higher than those in earlier instars (2nd~3rd), suggesting an enhanced digestive and metabolic potential to support the energy and material demands of rapid development. Additionally, winter low-temperature stress triggers a ROS burst and membrane lipid peroxidation within *J. przewalskii*, leading to the accumulation of lipid peroxidation end-products (e.g., MDA) in the cones [41]. This environmental stress may compel *M. sabinae* larvae to maintain high levels of GST expression as an antioxidant defense system during the prolonged overwintering period (~200 days in the 2nd instar), thereby clearing reactive oxygen species (ROS) generated by environmental stress and preventing oxidative damage to cellular components [32,42]. These strategies are likely shaped by the long-term, stable selective pressures of the high-altitude environment (low temperature, UV radiation and drought). Existing studies indicate that interactions between plants and their specialist herbivores can persist even under extreme conditions [43]. In this environment, overwintering survival constitutes the most critical life-cycle bottleneck for the larvae; consequently, any physiological adjustment that optimizes energy reserves (e.g., preserving lipids) and enhances antioxidant defenses (e.g., maintain high levels of GST) would be strongly selected for. Collectively, the *M. sabinae* larvae exhibit high plasticity in response to both host plant chemical defenses and high-altitude environmental stress, achieved through developmental-stage-specific regulation of the protein levels of key detoxification and digestive enzymes. This constitutes a potential adaptive molecular basis for their adaptation to the multi-layered defense system of *J. przewalskii*.

Leveraging LC-MS-based untargeted metabolomics, this study deciphered the multi-pathway coordinated defense network in infested *J. przewalskii* cones. Analysis revealed that differential abundant metabolites in infested endosperms were significantly enriched in the phenylpropanoid biosynthesis pathway, providing molecular-level corroboration for the observed PAL activity enhancement [44]. The sustained accumulation of flavonoids confers potent defensive functions. Flavonoid accumulation causes midgut damage in *Helicoverpa zea* larvae and disrupts the expression of key genes essential for maintaining gut health [45]. It also influences the oviposition behavior of *Papilio xuthus* on citrus juvenile leaves [46]. Coumarins, specifically enriched during the 2nd instar in this study (Figure 3a), can stimulate plant defense responses by triggering ROS generation and activating immune-related genes and signaling pathways (e.g., the salicylic-acid-dependent pathway). These compounds possess multifaceted anti-insect activities, including inhibiting various insect processes and hindering nucleic acid synthesis. This temporal alignment with the maintained high levels of detoxification enzymes, such as GST, in early instar larvae suggests a potential functional linkage between the two [47]. Such synchronized responses represent a key interactive mechanism ensuring synchronized development between the plant and insect. Furthermore, significant activation of the ABC transporter pathway unveils a spatial regulation mechanism in plant defense. For instance, transmembrane transport of secondary metabolites like terpenoids may be enhanced [48]. The novel enrichment of steroid derivatives during the 3rd~4th instar feeding period further supports this hypothesis, potentially facilitating the targeted accumulation of toxins at larval feeding sites [49]. Moreover, the abnormal activation of the retinol metabolism pathway raises the possibility that *J. przewalskii* may implement defense by, for example, disrupting insect molting hormone signaling [50].

In summary, this study systematically deciphers the intricate and sophisticated physiological interaction mechanisms between *J. przewalskii* and its specialist herbivore, *M. sabinae*. The highly specialized and tightly linked interaction between *J. przewalskii* and *M. sabinae* is likely a product of their long-term coevolution in adaptation to the extreme high-altitude environment. *Juniperus przewalskii* employs a comprehensive defense strategy involving the dynamic modulation of cone nutrient composition, the temporal activation of protective enzymes, and the construction of a multi-layered chemical defense network. Conversely, *M. sabinae* larvae exhibit remarkable adaptive capabilities, effectively countering host-imposed nutritional constraints and chemical challenges through the precise regulation and strategic division of labor among digestive and detoxification enzymes. This tight coupling of plant defense and insect counter-defense across temporal and metabolic dimensions likely represents an outcome of coevolution under the dual selective pressures of herbivory and the abiotic stresses (e.g., cold, drought) characteristic of the high-altitude habitat. This study not only provides novel perspectives for understanding the interaction mechanisms between oligophagous insects and their specific hosts but also contributes significant physiological and molecular evidence to the theory of plant–insect coevolution in alpine ecosystems. The interplay revealed here, although specific to the *J. przewalskii* and *M. sabinae* system, may represent a common physiological adaptation strategy within other *Megastigmus* and *Juniperus* associations, given their shared ecological context as specialist seed pests and their conifer hosts. Future comparative studies across different species pairs could test the generality of this model. However, it is important to note the methodological and contextual limitations of this study. First, our experimental design, where cones from multiple trees were pooled to create biological replicates, was aimed at characterizing the population-level average response of *J. przewalskii*. While this approach increases the statistical power to detect the core defensive response, it inherently integrates variation among individual trees and thus may underestimate the full range of phenotypic plasticity at the tree level, reducing the direct ecological generality of our findings to individual host responses. Second, this study was conducted under controlled laboratory conditions, omitting the influence of complex field variables such as temperature fluctuations and natural enemy interactions. Future research will integrate field experiments to validate the ecological applicability of these findings and delve deeper into the functional differentiation of detoxification enzyme genes and the interference mechanism of the host retinol pathway on insect hormone signaling. Furthermore, the findings of this study also point to potential directions for pest management based on similar plant–insect interaction systems, for instance, leveraging key induced defense compounds (e.g., specific coumarins or steroid derivatives) as natural product leads for developing novel biopesticides. These insights will furnish new evidence for the coevolutionary dynamics and bidirectional physiological adaptation mechanisms within plant-oligophagous pest systems in high-altitude ecosystems, laying a crucial foundation for developing ecologically sound pest management strategies based on host chemical defenses.

## Figures and Tables

**Table 1:** Correspondence between sampling dates and developmental stages of *Megastigmus sabinae* larvae.

Collection Date	Development Stage
25 August 2021	Egg stage
18 December 2021	2nd instar
5 April 2022	3rd instar
10 May 2022	4th instar
12 June 2022	5th instar

**Table 2:** Types of differential metabolites (DMs) categories and cumulative values of VIP scores in infested *Juniperus przewalskii* endosperms herbivory by *Megastigmus sabinae* larvae of the 2nd, 3rd and 4th instar.

Differential Metabolite Category	Type(s)	Cumulative Values of VIP Scores
Carboxylic acids and derivatives	9	34.90
Fatty acyl	10	34.09
Organic oxygen compounds	7	28.25
Flavonoids	6	17.19
Benzene and substituted derivatives	3	13.76
Keto acid and derivatives	3	10.20
Phenols	3	9.70
Purine nucleosides	3	9.67
Indole and derivatives	3	8.37
Imidazole pyrimidine	2	7.84
Prealcohol lipids	3	6.70
Benzoylpropionic acid	3	6.53
Pyrimidine nucleosides	3	6.49
Pregnenolone lipids	4	5.88
Steroids and derivatives	2	5.19
Hydroxyl acid and derivatives	2	4.97
Lactone	1	3.71
Nitrogen-containing organic compounds	2	3.41
Alcohols and polyols	1	3.33
Cinnamaldehyde	1	3.27
Purine nucleotides	2	3.18
Coumarin and derivatives	1	3.18
Furan lignans	1	1.93
Organic phosphoric acid and derivatives	1	1.88
Pyran compounds	1	1.78
Endogenous Metabolites	1	1.78
Pyridine and derivatives	1	1.75
Pteran dinitrogen heterocyclic compounds	1	1.71
Carbohydrate and its conjugates	1	1.70
Dinitrogen heterocyclic compounds	1	1.70
Triphenyl compounds	1	1.66
Alkaloids	1	1.66
Organic nitrogen compounds	1	1.58
Cinnamic acid and derivatives	1	1.53
Non-metallic oxyanion compounds	1	1.51
Diazobenzene	1	1.38

## Data Availability

The original contributions presented in this study are included in the article. Further inquiries can be directed to the corresponding author.

## Abbreviations

The following abbreviations are used in this manuscript:

ANOVA	Analysis of variance
CarE	Carboxylesterase
CYP450	Cytochrome P450 monooxygenases
DMs	Differential metabolites
ELISA	Enzyme-linked immunosorbent assay
GST	Glutathione S-transferase
KEGG	Kyoto Encyclopedia of Genes and Genomes
LC-MS	Liquid chromatography-mass spectrometry
PAL	Phenylalanine ammonia-lyase
POD	Peroxidase
QC	Quality control
ROS	Reactive oxygen species
VIP	Variable Importance in Projection
